# Characterization of Tunable Micro-Lenses with a Versatile Optical Measuring System

**DOI:** 10.3390/s18124396

**Published:** 2018-12-12

**Authors:** Sabina Merlo, Eleonora Crisà, Domenico Giusti, Marco Ferrera, Marco Soldo

**Affiliations:** 1 Dipartimento di Ingegneria Industriale e dell’Informazione, Università degli Studi di Pavia, 27100 Pavia, Italy; ecrisa@invensense.com; 2 STMicroelectronics, 20010 Cornaredo (Mi), Italy; domenico.giusti@st.com (D.G.); marco.ferrera@st.com (M.F.); marco.soldo@st.com (M.S.)

**Keywords:** lenses, microelectromechanical devices, micro-optics, optical interferometry, optical metrology, optical polymers, optical signal detection, piezoelectric actuators, piezoelectric film, thin film

## Abstract

In this work, we present the results of the opto–electro–mechanical characterization of tunable micro-lenses, Tlens^®^, performed with a single-spot optical measuring system. Tested devices are composed of a transparent soft polymer layer that is deposited on a supporting glass substrate and is covered by a glass membrane with a thin-film piezoelectric actuator on top. Near-infrared optical low-coherence reflectometry is exploited for both static and low-frequency dynamic analyses in the time domain. Optical thickness of the layers and of the overall structure, actuation efficiency, and hysteretic behavior of the piezo-actuator as a function of driving voltage are obtained by processing the back-reflected signal in different ways. The use of optical sources with relatively short coherence lengths allows performing interferometric measurements without spurious resonance effects due to multiple parallel interfaces, furthermore, selecting the plane/layer to be monitored. We finally report results of direct measurements of Tlens^®^ optical power as a function of driving voltage, performed by redirecting a He-Ne laser beam on the lens and monitoring the focused spot at various distances with a digital camera.

## 1. Introduction

Cameras in mobile devices are now extensively used for capturing sharp images, and commonly used autofocus mechanisms rely on electromagnetic voice-coil [1] or ultrasonic motors [2]. Micro-lenses with tunable focus length have recently been fabricated, exploiting microelectromechanical systems (MEMS) technology [3,4,5,6,7]. In particular, piezoelectrically actuated micro-lenses have been demonstrated, based on the deformation of a transparent fluid or soft polymer [8] placed between glass membranes or layers of other transparent materials. Compared to other current solutions, their main advantages consist in lower power consumption and faster response. Piezoelectrically actuated membranes are also the basic building blocks for other MEMS devices, such as micropumps [9,10].

For demonstrating the actual functionality of the fabricated devices, as well as for providing feedback to the design and process engineers, several parameters related to the opto–electro–mechanical properties of piezoelectrically tunable micro-lenses deserve to be directly measured. Here, we focus our attention on characterizing the properties of the piezoelectrically actuated Tlens^®^, developed by poLight and STMicroelectronics, which is composed of a polymer layer deposited on a supporting glass substrate, with a thin glass layer covering and a piezoelectric stack on top [8,11,12,13]. The thin glass membrane, and thus the underlying polymer, can be deformed by a voltage applied across the piezoelectric stack: a plano-convex lens is formed with voltage-tunable focal length. Detailed mathematical modeling of this device, also considering various geometries, is reported in [11,12,13]. The piezo-electric thin-film actuator was fabricated using the Thin-Film-Process (TFP) MEMS technology developed by STMicroelectronics [14,15,16,17].

MEMS testing can be realized using commercially available, but often costly and bulky, instruments; these techniques are usually quite powerful for looking at superficial static and dynamic features [18,19,20,21,22,23]. Other interferometric configurations were proposed to accurately measure the displacement of actuators and piezoelectric thin film [24,25,26]. In the case of a lens, however, it is interesting to also gain better insight into the inner structure, as it is an optical component acting on the light beam that is crossing its constituting layers. In this work, we present a versatile spot optical measuring system based on a flexible, compact fiberoptic configuration. This allows direct detection of the optical thickness of the layers and of the overall structure, and the hysteretic behavior of the piezo-actuator as a function of the driving voltage. After an initial alignment step to ensure that the lens in quiescent condition is perpendicular to the interferometric readout beam, then optical thickness, actuation efficiency (for displacement and optical thickness), and hysteresis can all be obtained by processing the back-reflected signal in different ways. Near-Infrared Optical Low-Coherence Reflectometry (NIR-OLCR) is the interferometric technique exploited for both static and low-frequency dynamic analyses in the time domain. 

In a previous work, we employed NIR-OLCR for detecting the displacement with hysteresis of the bare, thin glass membrane, driven by the piezoelectric thin film actuator (a subsection of the Tlens^®^) [27]. In this work, two fiber-coupled optical sources, both emitting in the NIR wavelength region but with different time-coherence, are utilized; the source with shorter coherence length for detecting interfaces of the inner layers, with enhanced longitudinal resolution, and the one with longer coherence length for monitoring ample optical thickness variations of the lens as a function of driving voltage. Finally, in this paper, we also report the direct measurement of the focal length f (or Optical Power, 1/f) of the Tlens^®^ as a function of the driving voltage, performed by redirecting a He-Ne (red) laser beam shined at 90° on the lens and monitoring the focused spot at various distances with a digital CMOS (Complementary Metal-Oxide Semiconductor) camera.

In Section 2, we briefly illustrate the structure and principle of operation of the Tlens^®^. In Section 3, the versatile instrumental configuration for opto–electro–mechanical characterization of micro-lenses by spot optical measurements is illustrated. After describing, in Section 4, how NIR-OLCR is exploited for structural characterization, we present the results relative to interferometric measurements of quasi-static lens displacement and optical thickness variations induced by piezo-actuation (Section 5). Finally, the results of direct detection of lens optical power as a function of dc driving voltage are illustrated in Section 6. Our experimental data confirm the Optical Power values estimated by means of numerical simulation based on the Finite Element Method and match the Optical Power values inferred by means of topographic analysis realized with a commercial vibrometer.

## 2. Piezoelectrically Actuated Tunable Micro-Lens

### 2.1. Structure

**Figure 1:**
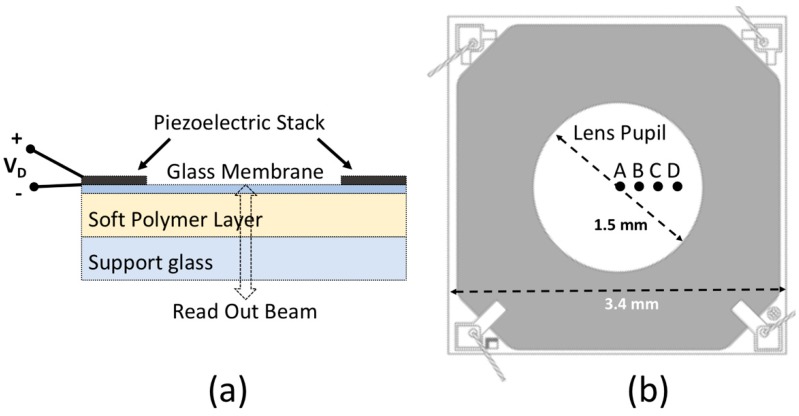
(**a**) Cross-sectional schematic structure of the Tlens^®^ in quiescent conditions. (**b**) Top view of the device showing the four radial positions (A, B, C, D) where measurements were performed. Sketches are not in scale.

Figure 1 shows the simplified structure of the cross section of a Tlens^®^, a micro-lens featuring a piezoelectrically tunable focal length. In particular, the devices tested in this work were developed by poLight ( Horten, Norway) and STMicrolectronics (Agrate Brianza, Italy) [28] and kindly mounted on a board by STMicroelectronics. The Tlens^®^ is a micro-optic component, realized with MEMS technology, which is formed by three optically transparent layers, aligned in quiescent conditions: 100-µm-thick glass support, 300-µm-thick soft polymer layer, 20-µm-thick glass membrane [8]. A ring-shaped piezoelectric actuator (1μm thick PZT53/47 thin film [14,15,16]) is fabricated on top of the thin glass membrane by thin film piezoelectric technology, leaving a circular transparent pupil at the center of the device with a diameter of approximately 1.5 mm. The device footprint is approximately less than 5 mm by 5 mm. 

The glass support and the glass membrane have different functions. The first, thick and rigid, supports the entire lens. The second, thin and flexible, bends along the borders by voltage driving the piezoelectric film and transfers its deformation to the underlying soft polymer layer [8].

### 2.2. Principle of Operation

When the piezoelectric film is not actuated (V_D_ = 0 V), it does not apply any force to the thin glass, which is approximately flat. When a positive driving voltage is applied between the top and bottom electrodes, the membrane and the underlying polymer layer are deformed and convex curved. The higher the driving voltage, the larger the structure deformation. The focal length of the plano-convex lens, modified by changing the curvature radius and the optical path-length or optical thickness, OT, of the structure, can be finely tuned by the driving voltage [11,12,13]. Note that the optical thickness is different from the geometrical thickness and is related to the actual path traveled by light, since OT = d × n_g_, where d is the geometrical thickness of the layer and n_g_ the group refractive index of the materials at the spectrum central wavelength of light traveling through the structure. Considering thick lenses (not just a thin lens approximation), the optical thickness keeps into account not only the effect of the geometrical thickness of the lens, but also the contribution of the refractive index of the material used for fabricating the lens. The diopter power of a lens is affected by the optical thickness, since the same diopter power can be achieved with a (geometrically) thicker lens but made of a material with lower refractive index, or with a (geometrically) thinner lens but made of a material with higher refractive index.

### 2.3. Opto-Electro-Mechanical Properties to Be Tested

Among the most interesting parameters that deserve to be directly measured for demonstrating the actual functionality of the fabricated device and for providing feedback to the design and process engineers, we have concentrated our attention on the optical thickness of the structure, on the actuation efficiency and the hysteretic behavior of the piezo-actuator, and on the focal length f (or optical power, 1/f) as functions of the driving voltage.

## 3. Instrumental Configuration for Opto-Electro-Mechanical Characterization of Micro-Lenses by Spot Optical Measurements

In the past we have demonstrated the great capabilities of semiconductor laser feedback interferometry for optical MEMS characterization with spot optical measurements [29,30,31], whereas in the case of the Tlens^®,^ we looked at a measuring technique that allowed to detect static as well as dynamic properties, with an instrumental configuration intrinsically capable of separating the contributions coming from the various layers forming the lens. Toward this aim, we selected optical low-coherence reflectometry operating in the near-infrared wavelength range (1.25 µm to 1.60 µm), previously described in [27,32,33], also known in the literature as white light interferometry [19,34,35]. This technique, implemented with an all-fiber set-up operating in the near infrared, allowed us to selectively monitor the electromagnetic fields back-reflected by the four dielectric interfaces, present along the light path that goes through Air-Glass Support-Polymer layer-Glass Membrane-Air. Interesting results of other authors on the performances of various interferometric techniques applied to MEMS testing can be found in [36,37,38,39].

**Figure 2:**
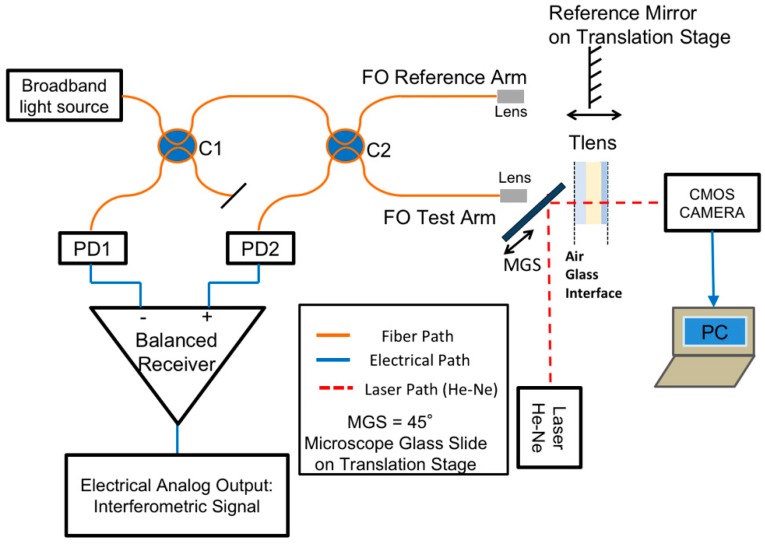
Reflectometric set-up. PD1, PD2: Photodiodes; C1, C2: 2 × 2, 50:50 fiberoptic coupler. FO—Fiberoptic path. MGS—Microscope Glass Slide on translation stage.

Figure 2 shows the assembled reflectometric set-up, consisting of a fiberoptic Michelson interferometer with balanced detector. By cascading a pair of fiberoptic bidirectional 2 × 2 splitters, both with a spectrally flat 50:50 splitting ratio, radiation from broadband light sources emitting in the selected near infrared wavelength range was guided along the “Test Arm” toward the Tlens^®^ and along the “Reference Arm” toward the reference mirror. More details on the employed optical components were reported in [27,32,33]. A balanced receiver, with InGaAs photodiodes, achieved efficient visualization of the interferometric fringes that were generated when the relative difference between the optical path-length of the reference and test arms was changed. Using a broadband optical source, interferometric fringes were developed and were clearly detectable when this difference was not larger than the coherence length of the readout radiation. This feature allowed measuring of the optical path-length, or optical thickness, of the lens layers (for static characterization) by displacing the reference mirror as well as monitoring the displacement or the optical path-length variations induced by the driving voltage, with subwavelength resolution typical of interferometric methods. Moreover, the implemented scheme was suitable for measuring the components of displacement or optical path-length along the direction of the impinging read-out light beam, which had a neglectable divergence along the considered working distance and was oriented orthogonally to the lens in quiescent condition. The limited time coherence of the readout source, considered a drawback in laser interferometers, becomes very useful when testing multilayer planar structures incorporating multiple parallel interfaces. It allows detection of the displacement of a selected plane/layer without spurious interfering effects, due to back-reflection contributions coming from other interfaces but traveling all along the same path. Once the lens in quiescent condition was aligned perpendicularly to the interferometric test beam, so that back coupling of reflected light from interfaces was optimized, it was possible to directly measure the focal length without jeopardizing the previous alignment by redirecting the red (0.63 µm wavelength) collimated beam (provided by a He-Ne laser, Siemens, Munich, Germany), shined at 90°, just by inserting a glass slide at 45° in front of the Tlens^®^.

## 4. OLCR for Device Structural Characterization

**Figure 3:**
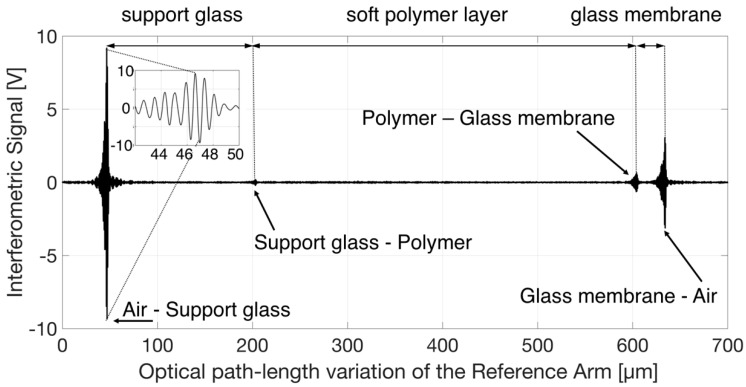
Interferometric signal as a function of the optical path-length variation of the Reference Arm. Four groups of amplitude modulated fringes can be recognized. Inset: zoom of the signal that shows detail of the amplitude-modulated fringes.

The structural characterization of the Tlens^®^ consisted of detecting the optical thickness, or optical path-length, of each single layer, in a non-invasive and non-destructive way, so that the measurements could be performed multiple times without any damage to the lens. For accomplishing this task, we employed a Tungsten lamp, an ultra-broadband read-out source previously exploited for the structural testing of other devices [32,33]. Although the fiber-coupled optical power was low, its short coherence length, of the order of a few microns, ensured a suitable longitudinal (or in-depth) resolution for selectively detecting the relative position of the inner interfaces, despite the low field reflectivity caused by the small refractive index step among glass and polymer. By longitudinally translating the reference mirror (at constant velocity v = 10 µm/s with a computer controlled, motorized linear translation stage), the optical path-length of the Reference Arm was increased. The analog output of the balanced receiver was digitally converted, acquired in the time domain by a personal computer and then translated into optical path-length variations. Amplitude-modulated, interferometric fringes were observed when the Reference Arm length matched the optical path-length on the Test Arm up to each interface within ±2 µm. A typical result of this scan is reported in Figure 3, which shows the acquired interferometric signal as a function of the optical path-length variation of the Reference Arm. Four groups of fringes, amplitude-modulated by the fringe visibility function that mainly depends on the line-shape of the emission spectrum of the source, can be recognized. Moreover, the envelopes of each fringe group exhibit different peak amplitudes. The group with the highest peak-amplitude is due to the signal being back-reflected by the first crossed interface between air and support glass. Another high peak is related to back-reflection coming from the interface between glass membrane and air. Whereas the glass membrane has an anti-reflection dielectric coating for visible light, it does not prevent reflection of near-infrared light. The interfaces between glass and polymer provide back-reflected signals that generate two separate groups of fringes with much lower peak amplitude, which is expected due to the similar refractive indices of these materials. The envelope peak amplitude of the last fringe group is lower than the envelope peak amplitude of the first fringe group, though it is an air–glass interface, due to the fact that the fraction of the field amplitude reflected by the last interface and coupled back into the readout fiber is lower than that reflected at the first air–glass interface. There are optical losses at each interface, due to reflections and refractions. Moreover, the back-coupling efficiency on both the Reference and Test arm, at the optical distance corresponding to the further glass–air interface, becomes lower due to the limited working distance of the aspheric lenses placed on the tip of the readout optical fibers. Although we do not know the exact values of the refractive indices of both glasses, we know that the thin glass membrane has a higher refractive index than that of the supporting glass. The optical thickness of the various layers measured in the center of the Tlens^®^ was then obtained as the optical distance between the groups of fringes. As a matter of fact, the position of the peak is a precise indicator of the moment when the interferometer paths are equal for any given reflection. By relating the position of the peak with the position of the Reference arm, the separation of the interfering reflection can be determined. The following values for the OTs can be estimated from the reported data: OT of Support Glass is 156 µm, OT of Polymer layer is 400 µm, OT of Doped Glass is 30 µm. To the best of our knowledge, the group refractive indices of the materials composing the various layers are in the range 1.5–1.6 RIU (Refractive Index Unit). Thus, remembering that OT = n_g_ × d, where n_g_ is the group refractive index and d the geometrical thickness, assuming for n_g_ an intermediate value, we obtain that the support glass geometric thickness is of the order of 100 µm, the polymer layer thickness is of the order of 257 µm, and the glass membrane geometric thickness is of the order of 19 µm. The inset in Figure 3 reports a zoom of the signal that shows the amplitude-modulated fringes. The distance between two consecutive minima or maxima corresponds to an optical path variation of λ_c_/2, where λ_c_ is the center emission wavelength of the read-out source, in this case λ_c_ = 1.57 µm.

## 5. Quasi-Static Lens Displacement and Optical Thickness Variations Induced by Piezo-Actuation

A second important aim to achieve for Tlens^®^ characterization consisted of detecting the displacement of the lens glass-support and, even more importantly, the lens optical thickness (OT) variation as a function of the voltage applied to the piezo-actuator in quasi-static conditions. For this purpose, the piezo-actuator was driven with an AC voltage consisting of a triangle wave at 45 Hz, from 0 V up to +40 V, which is the whole dynamic range of the recommended driving voltage. Since both displacement and OT variations were expected to be larger than a few µm, the coherence length of the Tungsten lamp was too short. For these measurements, the selected broadband read-out source was a fiber-coupled Superluminescent Light Emitting Diode (SLED, Covega Thorlabs SLD1021, Newton, NJ, USA) with a Gaussian emission spectrum centered at λ_c_ ≈ 1351 nm and Full Width at Half Maximum bandwidth (FWHM) ≈ 52 nm. Radiation provided by this source exhibited a coherence length in free space L_c_ ≈ 20 µm, short enough to discriminate the various interfaces but at the same time long enough to provide interferometric signals with an excellent signal-to-noise ratio, even for the length mismatch between the Reference and Test Arm up to ±10 µm. Propagation of the fundamental mode in the fiberoptic path was also ensured in the SLED emission bandwidth thanks to the 1250 nm cut-off wavelength of fiberoptic patch cables and components.

**Figure 4:**
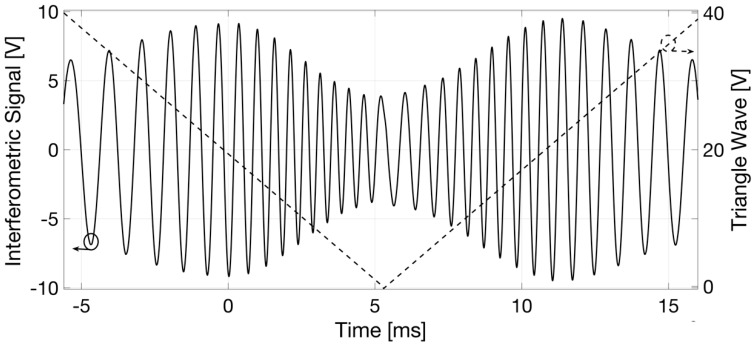
Interferometric signal (black continuous trace, left axis) and triangle driving wave (black dashed trace, right axis) as a function of time. The interferometric fringes appear amplitude modulated by a gaussian visibility function, as expected using the SLED due to its Gaussian emission spectrum.

The interferometric signal and triangle driving wave in the time domain were acquired with a digital oscilloscope, and both signals are plotted as a function of time in Figure 4. The interferometric fringes (black trace) appear to be amplitude modulated by a gaussian-like visibility function, as expected using the SLED. To achieve this result, the reference mirror position was selected and fixed in order to attain the peak of the fringe group relative to the air-support glass interface (thus, the closest to the reading-fiber termination) at approximately half of the driving voltage ramp.

**Figure 5:**
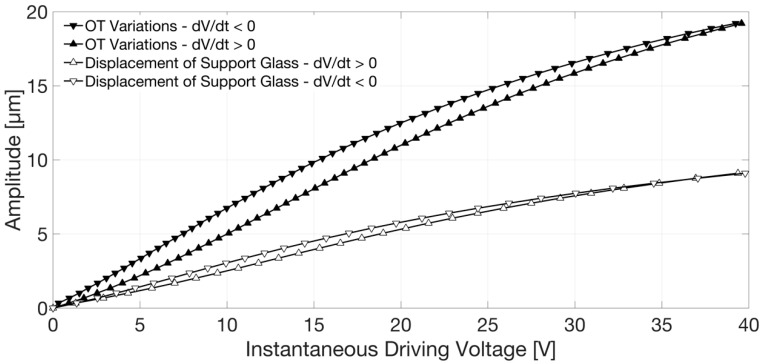
Amplitude of the displacement (white filled markers) of the support glass and of the optical thickness (OT) variations (black filled markers) as a function of the instantaneous driving voltage. △: Displacement for dV/dt > 0. ▽: Displacement for dV/dt < 0. ▲: OT for dV/dt > 0. ▼: OT for dV/dt < 0.

The displacement of the support glass was finally reconstructed as a function of the instantaneous value of the piezo-actuator driving voltage by fringe counting with a λ/8 = 169 nm resolution, that is by detecting the voltage corresponding to all the maxima, minima, and zero crossings of the interferometric signal. The result is presented in Figure 5. Hysteresis in the position assumed by the glass support, with respect to the starting condition, for increasing and decreasing voltages, is clearly visible in this graph as the trace relative to the amplitude of displacement obtained for dV/dt > 0 (white filled markers △) is not superposed to the trace relative to the amplitude of displacement attained for dV/dt < 0 (white filled markers ▽). The detected displacement and the hysteretic cycle were found in agreement with the results provided by numerical simulations [14,26,27]. Similar hysteretic behaviors, not strictly related to the device shape and structural materials, were reported by other authors in [9,10,25].

**Figure 6:**
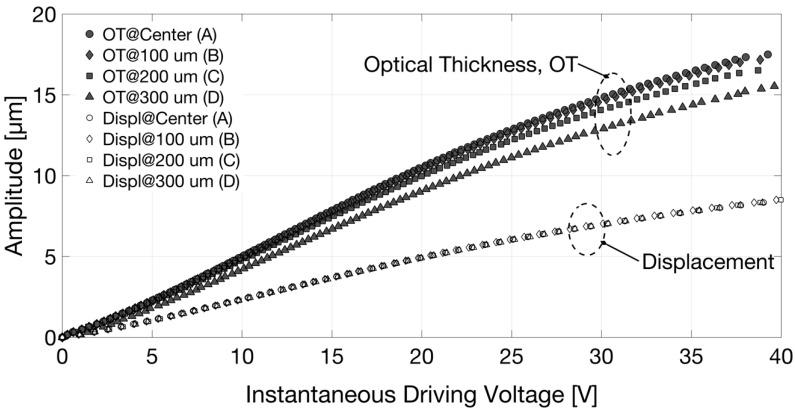
Amplitude of the displacement (Displ) (white filled markers) of the support glass and of the optical thickness (OT) (black filled markers) variations as a function of the instantaneous driving voltage, for dV/dt > 0, in positions A (pupil center), B, C, and D (at increasing distances from the lens pupil center). Circles ●○: results at pupil center (position A). Diamonds ◆◇: results @100 µm (position B). Squares ■□: results @200 µm (position C). Triangles ▲△: results @300 µm (position D).

As the diameter of the readout beam in the infrared is approximately 50 µm, the spatial resolution could be quite good: spot optical measurements performed in a few positions could provide data on the device deformation. Figure 6 shows the displacement measured for dV/dt > 0 in the 4 radial positions, specified in Figure 1b; the displacement of the support glass was the same in all the tested positions. 

A better insight into lens tunability was obtained by detecting the lens optical thickness (OT) variations as a function of the voltage applied to the piezo-actuator. It is important to emphasize once more that the optical thickness is different from the geometrical thickness of the layer, as it is defined as OT = d × n_g_, where d is the geometrical thickness of the layer and n_g_ the group refractive index at the center emission wavelength of the source.

For this investigation, the reference mirror position was selected and fixed in order to attain the peak of the fringe group relative to the interface between glass membrane and air, thus the furthest position away from the fiber termination, with approximately half of the driving voltage peak. The interferometric signal and triangle driving wave in the time domain were also acquired in this case with a digital oscilloscope; the reconstructed optical thickness variation as a function of the instantaneous driving voltage is reported in Figure 5, directly compared with the displacement of the support glass previously described. The amplitude of OT obtained for a given voltage for dV/dt > 0 (black filled markers ▲ in Figure 5) is different from the one attained for the same applied voltage, but dV/dt < 0 (black filled markers ▼ in Figure 5). OT variations are much larger than the displacement of the support glass due to the soft polymer deformation. Moreover, they assumed smaller values moving the spot of the readout beam from the center towards the border of the lens pupil (in the positions indicated in Figure 1b), as shown in Figure 6, due to the curved shape of the induced deformation (as expected for a convex lens). It should be noted that when considering only the glass membrane with the piezo-actuator, thus without the polymer layer and the support glass, the displacement obtained by monitoring the air–glass front interface did not differ from the OT variations measured by monitoring the glass–air back interface. In that case, the optical thickness change of the glass layer induced by the thin-film piezo-actuator was well below the resolution of the implemented method.

Once the displacement of the support glass as a function of the applied voltage, Disp(V), or the OT variations as a function of the applied voltage, OT(V), have been obtained from the interferometric measurements, one can obtain the actuation efficiency by taking the derivative of these relationships. We can define the efficiency parameters E_OT_, as E_OT_ = d[OT(V)]/dV, and E_Disp_, as E_Disp_ = d[Disp(V)]/dV. Since the functions Disp(V) and OT(V) are not linear, we cannot find just an efficiency value that is valid for all applied voltage. From the data collected for increasing voltage, we found a maximum value E_OT,Max_~0.6 µm/V and E_Disp,Max_~0.28 µm/V.

## 6. Setup Reconfiguration for Direct Detection of Lens Optical Power as a Function of DC Driving Voltage

To complete the lens characterization, it was also necessary to add to the set-up the capability for focal length direct detection as a function of the applied voltage. Toward this aim, we introduced a 45° Microscope Glass Slide (MGS in Figure 2) on a translation stage between the termination of the Test Arm and the Tlens^®^. For the sake of compactness, the collimated laser beam provided by a red-emitting He-Ne laser was introduced askew and bent by the 45° MGS. When we exploited low coherence reflectometry, the He-Ne laser was off and the MGS was pulled out; when we performed focal length measurements instead, the readout broadband source was off and the MGS was brought forward. Focal length detection was thus achieved without compromising the alignment of the reflectometer on the device under testing. This option could be quite interesting during reliability studies for investigating, for example, aging effects, when repeated measuring sessions of all lens features need to be carried on.

The lens was actuated by applying a DC voltage by means of a power supply, and always driven with increasing values of voltage: because of the hysteresis, the required voltage for focusing light at a certain distance would be different if it was reached by increasing or by decreasing the driving voltage. Once the CMOS camera was placed in a predefined position, the aim was to find the DC voltage value to apply to the lens in order to obtain the spot on the camera with the highest intensity. We selected 13 positions for the camera to be placed, with distances from the lens ranging from 11.6 cm up to 11.2 m, in order to characterize the lens behavior in a sufficiently wide range of focal lengths. The measurements were performed in the dark in order to avoid any light contribution, other than that of the laser beam, which would have perturbed the reading of the CMOS camera. 

**Figure 7:**
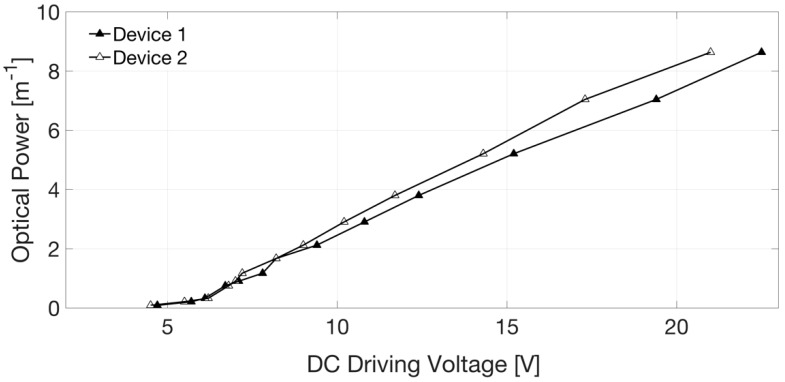
Optical power as a function of the DC driving voltage applied to the piezo-actuator. Results obtained for two different Tlens^®^ devices.

**Figure 8:**
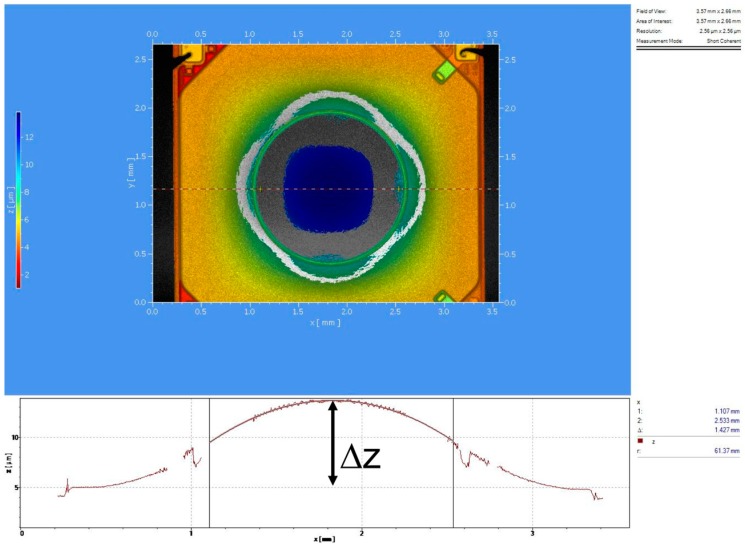
Results of a topographic measurement performed with a Polytec Micro-System Analyzer MSA 500 on a Tlens^®^ actuated with 20 V. On the z-axis, in false colors is height reached by the deformed surface of the lens. The maximum height variation in the center of the pupil is Δz ~ 8.8 µm.

**Figure 9:**
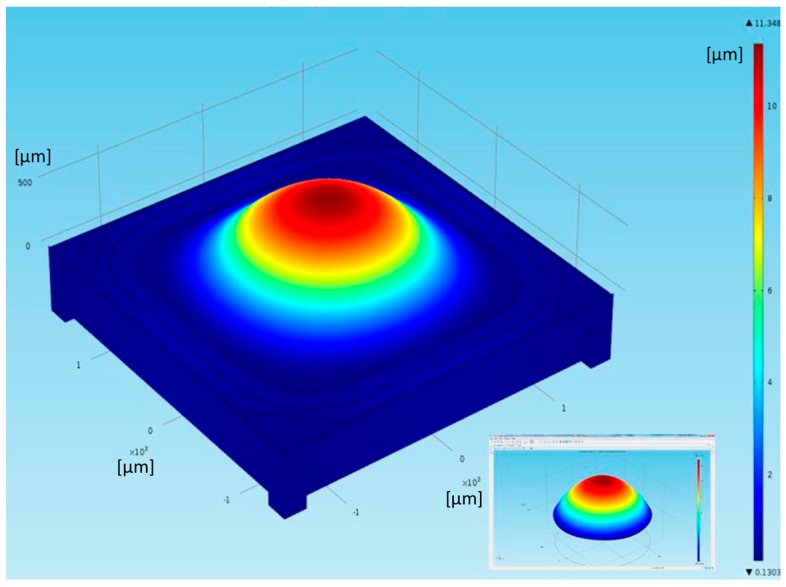
Results of a numerical simulation, based on Finite Element Method using COMSOL™, which predicts the convex shape assumed by the lens when the piezo-actuator is driven with 30 V. On the right, the false color scale for the height reached by the deformed surface of the lens is shown. The maximum height variation in the center of the pupil is Δz = 11.348 µm.

The camera software allowed monitoring of the intensity of the laser spot on the CMOS sensor and identification of the approximate range of DC voltage that contained the focusing value. Pictures were usually acquired for 10 increasing voltage values in this range, with 100 mV steps. These images were then elaborated through a MATLAB code to find the one with the maximum intensity value; the corresponding applied voltage was the closest value (with an error of ±50 mV) to the required driving voltage to focus the input collimated beam in the selected position. Figure 7 shows typical results of these measurements and data analysis, depicted in terms of Optical Power in m^−1^ (or diopter) as a function of the applied voltage. Experimental values, collected with our direct method, were compared with the values calculated by processing the curvature radius of the lens surface provided by topographic measurements with a Polytec Micro-System Analyzer MSA 500 (a laser vibrometer) by Polytech, Waldbronn, Germany. A typical result of this kind of testing obtained on a different sample of Tlens^®^ driven with 20 V is shown in Figure 8. On the z-axis, in false colors, the surface height is shown. The maximum height variation in the center of the pupil is Δz ~ 8.8 µm. From the section line shape, the curvature radius is calculated. Exploiting the lens maker formula, thus with an indirect method, we found (for 20 V driving voltage) a dioptric power of 8 m^−1^ that is in accordance with the results shown in Figure 7. Moreover, all experimental data confirmed the Optical Power values estimated by means of numerical simulation based on Finite Element Method using COMSOL™ with a fully coupled piezo-electromechanical modeling interface. An example of the output of these simulations is reported in Figure 9, which shows the predicted convex shape assumed by the lens when the piezo-actuator is driven by 30 V. The maximum height variation in the center of the pupil is Δz = 11.348 µm. From the curvature assumed by the lens foreseen by the numerical tool, we could indirectly assess an Optical Power of 3 m^−1^ for a driving voltage of 10 V, an optical power of 8 m^−1^ for a driving voltage of 20 V, and an optical power of 11 m^−1^ for a driving voltage of 30 V. Results of our direct measurements of Optical Power as a function of the driving voltage were also in general agreement with calculated values reported by other authors in [12].

## 7. Conclusions

We have reported the results of the experimental characterization of Tlens^®^ tunable micro-lenses by means of a versatile spot optical measuring system. A few samples were investigated to show device performances and potential of the measuring configuration. Comparisons of our results with expected design data was mainly qualitative since fine details relative to the characteristic dimensions of the device were only approximately known. Future work will be devoted to directly confronting the expected performances obtained by numerical modeling and calculations with the experimental results on fabricated devices with well-known geometric parameters.
